# Optimization and experimental analysis of multi-path sweet potato transplanter device

**DOI:** 10.1038/s41598-025-90732-7

**Published:** 2025-04-01

**Authors:** Zhengduo Liu, Wenxiu Zheng, Zhaoqin Lv, Junqing Pan

**Affiliations:** 1 College of Mechanical and Vehicle Engineering, Western Anhui University, Lu’an City, 237000 China; 2 Shandong Provincial Key Laboratory of Horticultural Machinery and Equipment, Tai’an City, 271018 China

**Keywords:** Agricultural machinery, Transplanter, Sweet potato seedlings, Transplanting robot, Field trials, Mechanical engineering

## Abstract

The low accuracy of the tracks is crucial problem in the development of the sweet potato industry. This paper optimizes a previously designed multi-track transplanting device for the multi-track transplanting of potato seedlings. The performed optimization is based on theoretical analysis, DEM–MBD coupling simulation, and field test, allowing to increase the accuracy of potato seedling transplanting. Kinematic analysis of the transplanted robotic arm is conducted to combine the single-factor test with the reference trajectory size characteristics. To study the impacts of the seedling claw length and initial angle on the transplanting trajectory, the following optimal parameter combination is determined: rod length of 0.4 m and initial angle seedling claw of 35°. A many-body kinetic model of transplanting is developed using the DEM–MBD coupled simulation method. Field experiments are then conducted to study the impact of the soil particles on the transplanting mode and position change of potato seedlings. In these experiments, an operation speed of 0.2 m/s and a seedling leakage length of 50 mm are adopted. The obtained results show that the qualified rates of oblique and horizontal transplanting are 96.8% and 96.9%, respectively. This study provides a reference for the development and optimization of sweet potato transplanting machinery.

## Introduction

Transplanting is a critical stage in sweet potato production. It involves labor accounting for approximately 23% of the entire production process^1–3^. The mechanical transplanting technology for sweet potatoes was launched in the 1950s. It then undergone a slow progress, yielding few practical transplanting tools. Manual planting has been commonly used for a long time. However, it requires high labor intensity, which results in low transplanting efficiency. In recent years, the structural shortage of labor in rural areas resulted in the high demand for sweet potato transplanting machinery. The lack of such technology significantly limited the development of the sweet potato industry^4–6^.

The method of sweet potato planting significantly affects the growth, quality, and yield of potato seedlings, that determine the planting benefit^7^. The existing sweet potato planting methods include the straight transplanting, oblique transplanting, horizontal planting, and boat bottom planting. Due to the lack of transplanting equipment, the sweet potato transplanting still mainly relies on manual labor^8–11^. In the United States, large chain-type bare root transplanters dominate the market, such as the large semi-automatic chain-type sweet potato transplanter manufactured by the American Marcinick Company, which can simultaneously transplant sweet potatoes in more than eight rows. In the operation process, high-power tractors are used for traction, manual separation of seedlings is conducted, and watering is performed after transplanting^12^. Afterwards, the Sevillia University added support to the chain and optimized the transplanting trajectory, which allowed to improve these chain-type transplanters. However, the latter can only be used for direct transplanting, and they may damage the ridges during operation^13^. Many countries, including Japan, mainly use small-scale transplanters, such as the 2ZYZ-2 small self-propelled transplanter produced by the Toyo-Kan Company, which allows to adjust the transplanting angle and depth. This transplanter has been widely used due to its simple structure and stable results^14^. Hu et al.^15^ developed the 2ZGF-2 sweet potato compound transplanting machine, which is able to simultaneously perform ridge creation, transplanting, and compaction for two rows of sweet potatoes. It is also able to modify the transplanting depth of the sweet potatoes by adjusting the lifting rod connected to the tractor. This machine performs inclined transplanting, and it led to satisfactory results in many application domains. Wu et al.^16^ designed a seedling belt for the fixation of sweet potato naked seedlings, and developed the corresponding seedling delivery mechanism. This device solves the efficiency bottleneck caused by artificial seeding in the transplanting process, and separates the seeding operation and transplanting operation to improve the transplanting efficiency. However, when the sweet potato seedlings are installed on the seedling belt, it still needs to rely on manual seeding installation, and the actual labor force required in the overall transplanting process has not been improved.

Mu et al.^17^ considering the lack of sweet potato horizontal transplanting solutions available in the market, focuses on the agronomic requirements for sweet potato horizontal transplanting. This study conducts theoretical research on the movement of sweet potato horizontal duplex transplanting machines, simulates and analyzes the main structural parameters of the planting claws, and carries out experimental research. This work provides a reference for the theoretical research and innovative design of sweet potato mechanization in horizontal transplanting.

The existing sweet potato transplanters mainly have two limitations:

1. Low integration of agricultural machinery and agronomy

The traditional sweet potato transplanting devices have only one degree of freedom, which is achieved through the rotation of the active component and a complex mechanical structure for the establishment of a set transplanting trajectory. In addition, it is challenging to ensure that the transplanting trajectory totally meets the agronomic requirements, which results in restricting the development of the mechanized sweet potato transplanting.

(2) Limited diversity in mechanized transplanting methods

The existing sweet potato mechanized transplanting methods mostly involve direct or inclined transplanting, while the boat-shaped and horizontal transplanting are not mechanized yet. The limited diversity in seedling transplanting methods and the associated low work quality constrain the further development of the sweet potato industry. To solve these problems, this study develops a sweet potato transplanter able to implement different transplanting methods. This allows to enhance the single transplanting method and increase the work quality in sweet potato cultivation.

The robotic arm has compact structure and flexible movements, which require minimal power and space. In addition, when combined with various sensors, it can complete complex transplanting trajectories^18–22^. Many countries, such as the Netherlands and Japan, developed robots for greenhouse farming tasks, including harvesting, weeding, and spraying for various crops (e.g., cucumbers, tomatoes, and cabbages^23–25^). Yin et al.^26^ first developed an articulated fruit and vegetable harvesting robotic arm. They then optimized its structural parameters through a case study incorporating the application of cucumber harvesting operations in greenhouse environments. Furthermore, they conducted many experiments demonstrating that this optimized robotic arm can adequately cover all the necessary target spaces for cucumber harvesting tasks, which meets the operational demands. … et al.^27^ and … et al.^28^ developed a kiwifruit harvesting robot equipped with four robotic arms. They then conducted many field trials using this robot. Their results demonstrated that their proposed configuration leads to stable and efficient harvesting results, and thus it can partly replace the manual labor. Xiang et al.^29^ proposed a phalanx-type underactuated picking mechanical claw featuring two degrees of freedom per joint. During picking, suction cups at the center of this mechanical claw grasp apples, while a retractable cutting blade severs their stems. Pressure sensors are embedded in the inner wall of the fingers to prevent the excessive pressure that may damage the apples. Although the aforementioned transplanting equipment led to satisfactory results, to the best of our knowledge, there is no special mechanical arm transplanting equipment for crops that operate outdoors (e.g., sweet potatoes), whose transplanting trajectory significantly affects the yield and survival rate.

To solve the aforementioned problems, this study incorporates agronomic practices related to sweet potato cultivation into existing approaches. This allows to further optimize the sweet potato transplanting mechanism. This provides theoretical guidance and technical support to tackle many related challenges, including the limited diversity in the mechanized sweet potato transplanting methods and the low integration of agricultural machinery and agronomy.

Thus, based on a previous study, and taking the potato seedlings transplanting agronomy requirements into consideration, theoretical analysis, DEM–MBD simulation, and field experiment, are conducted to further optimize the mechanical arm sweet potato transplanting. This makes the machine control the transplanting trajectory and follow various transplanting ways, according to the agronomy requirements. Consequently, the labor efficiency of potato farmers and the plant survival rate are improved. This study provides practical machines and tools for the sweet potato planting market, as well as a reference for the development and optimization of sweet potato planting machinery.

## Transplanter structure and key parts design

### Structural and working principle of the transplanter

**Fig. 1:**
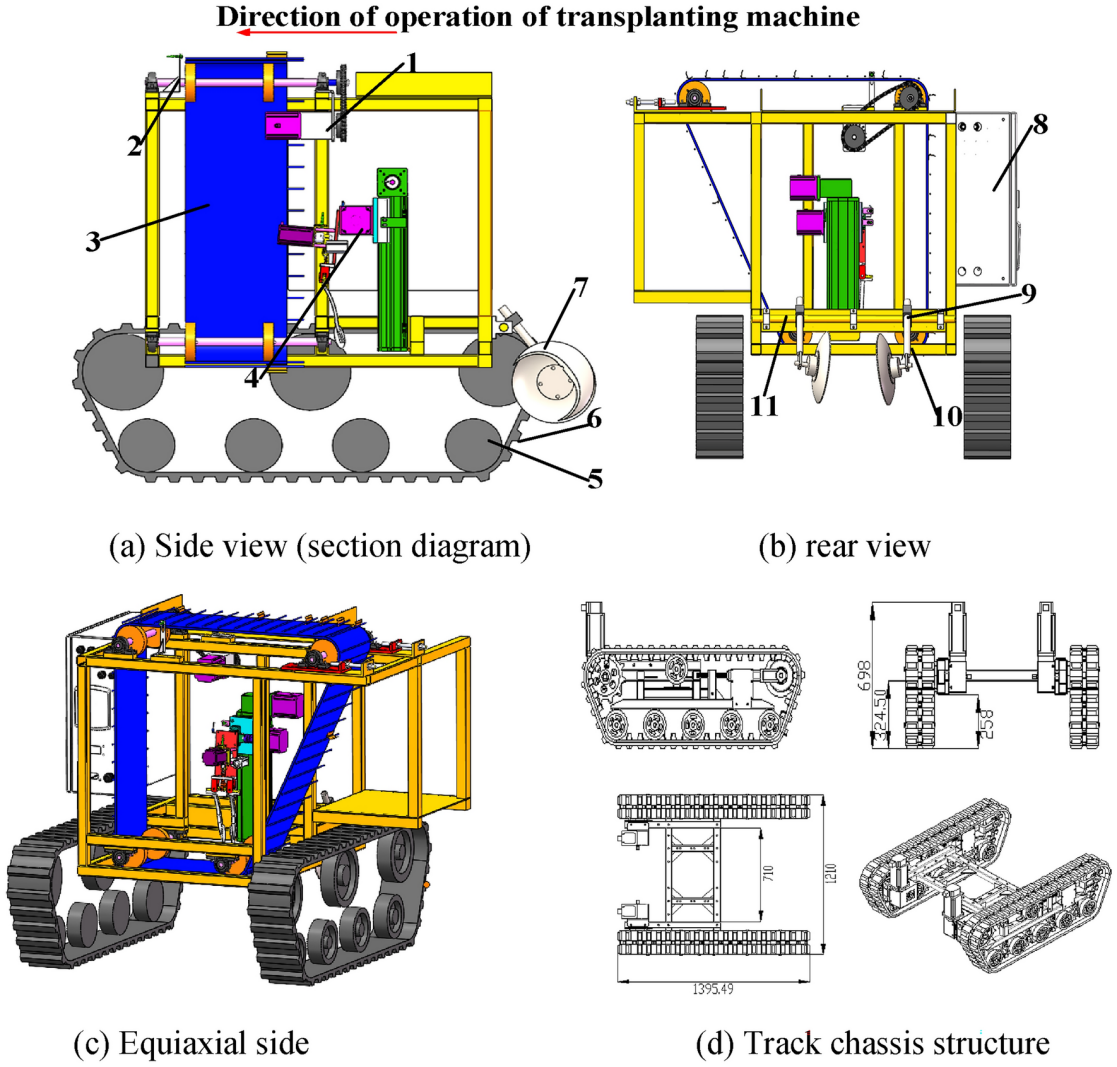
Structural diagram of the sweet potato transplanter. 1. Potato conveyor belt drive motor 2. Potato conveyor belt proximity switch 3. Potato transmission mechanism 4. Transplanting arm 5. Roadwheel 6. Track 7. Cover disc 8. Control box 9. Cross optical shaft connector 10. Horizontal shaft 11. Vertical shaft.

The overall structure of the machine is illustrated in Fig. 1. It incorporates the design of a mechanical arm-type sweet potato transplanter, which is convenient for large ridge single-row transplanting mode in modern sweet potato industry transplanting base.

The developed sweet potato transplanter mainly comprises four parts: transplanting device, crawler chassis, covering mechanism, and control box. The transplanting device consists of a mechanical arm and a seedling conveyor used to transfer the sweet potato seedlings. The crawler chassis consists of tracks and support wheels. The covering mechanism consists of horizontal axis, covering disc, vertical axis, and cross light axle connector. The control box consists of the controller of the transplanting device and a laptop. The crawler chassis accepts the weight of the entire transplanter while traveling in furrows. It also sends the travel distance to the transplanting machine controller to ensure a proper spacing between all the sweet potato plants. To secure the seedlings, the covering mechanism fills the holes formed by the digging claws with soil.

### Design of the transmission mechanism

**Fig. 2:**
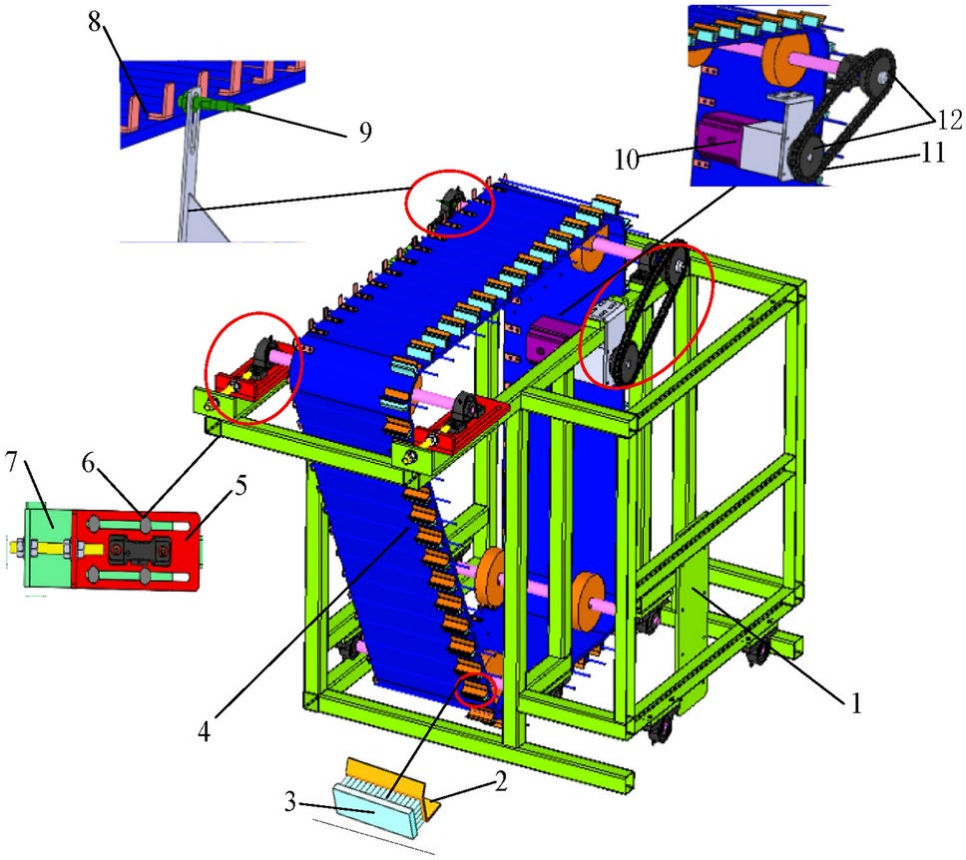
Sweet potato seedling transmission mechanism. 1. Fixing plate 2. Baffle plate 3. Brush 4. Sweet potato seedling conveyor belt 5.Slide plate 6. Tighten screws 7. Slide board 8. Inductive metal 9. Proximity switch of sweet potato seedling conveyor 10. Stepping motor 12. Chains.

Figure 2 shows the sweet potato seedling transmission mechanism, which comprises a stepping motor, a sweet potato seedling conveyor belt, and a sweet potato seedling fixing device^30^. The sweet potato seedling conveyor belt and fixing device are equipped with induction metal and proximity switch, respectively. The controller determines if the sweet potato seedlings are transported to the transplanting position through the electric signal returned by the proximity switch.

The traditional transplanter usually adjusts the power transmission direction and transmission ratio through transmission gears. This allows it to accurately pick up and transplant sweet potato seedlings according to the preset plant distance. However, its mechanism is complex and the plant distance cannot be adjusted based on the actual requirements. In this paper, the chassis, transmission mechanism, and transplanting mechanism of the proposed transplanting machine are independent. The controller receives the position of each machine through the proximity switch, which allows to simplify the coupling between the mechanisms and to adjust the transplanting distance based on the actual requirements.

### Design of the soil covering mechanism

**Fig. 3:**
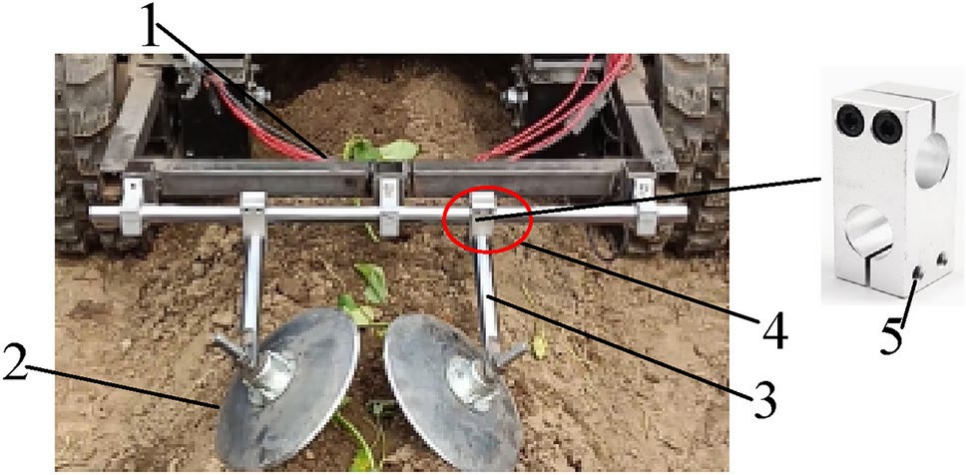
Structure diagram of soil covering mechanism 1. Horizontal axis 2. Soil cover disc 3. Vertical axis 4. Cross beam connector 5. Tightening screw hole.

The soil covering mechanism is composed of shaft fixed seat, horizontal shaft, earth covering disk, vertical shaft, and cross optical axis connector, as shown in Fig. 3. The axle fixing seat fixes the horizontal axis on the frame. In addition, the cross optical axis connector is set on the other end of the horizontal axis to link the vertical axis with the vertical shaft. The distance between the disks of the soil can be adjusted by tightening the screws according to the width and height of the ridge.

**Fig. 4:**
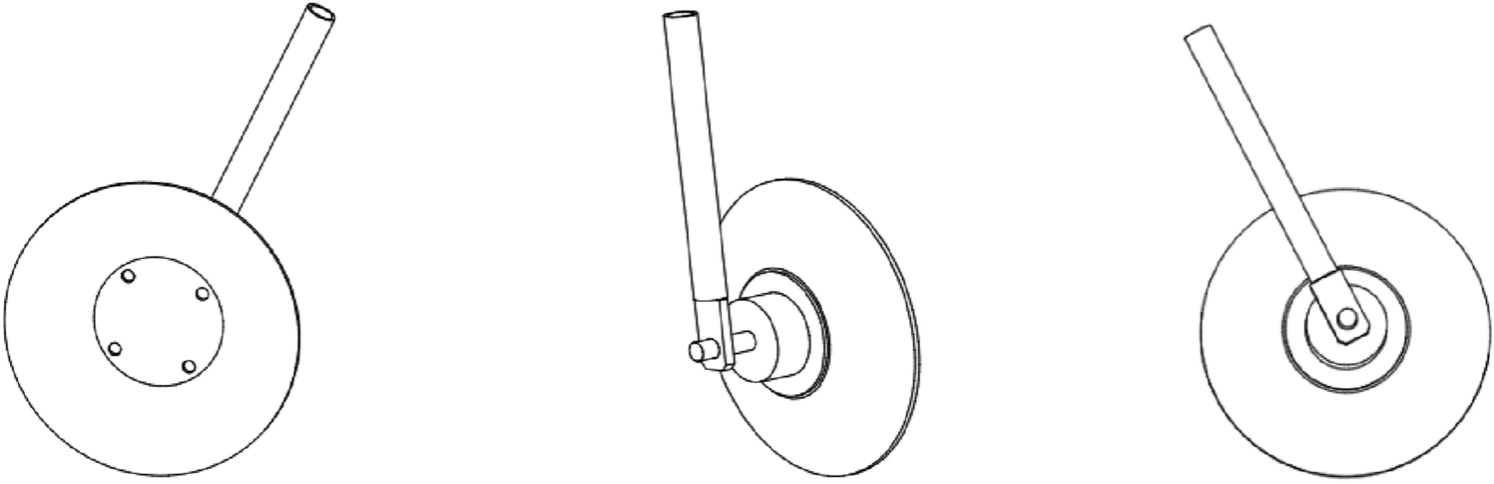
Three-dimensional model of soil-covered disk (**a**) left view (**b**) axonometric view (**c**) right view.

The soil covering disc gathers broken soil and fill pits. Its performance significantly affects the soil covering quality. According to the technical procedures, the cross-section of the sweet potato covered soil is rectangular with a cross-sectional area not less than 5 cm × 2 cm. The contour shape of the disc is spherical, its diameter is equal to 320 mm, the angle between its direction and the unit is equal to 20°, the installation angle is equal to 35°, and the cutting effect is good. When the spacing between the two discs is set to 0.38 m, the design parameters can meet the requirements of the sweet potato transplanting machine. The 3D model of the disc is shown in Fig. 4.

### Design and optimization of the transplanting machine arm

#### Structural design

**Fig. 5:**
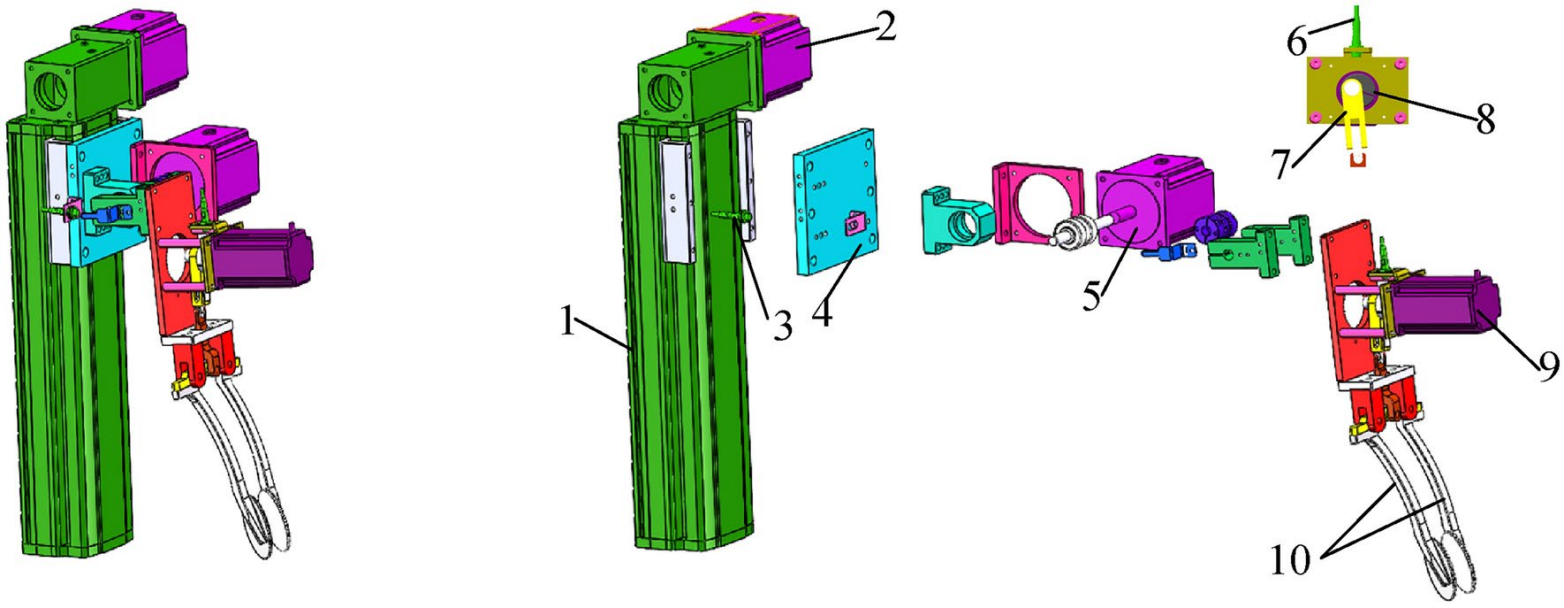
Sweet potato transplanting robot arm (**a**) Isometric view (**b**) Explosion view. 1. Sliding plate 2. Sliding plate motor 3. Proximity switch 4. Seedling claw fixing plate 5. Rotating motor 6. Proximity switch 7. Adjustment screw 8. Eccentric gear 9. Seedling claw motor 10. Seedling claw.

This article improves the structure of the transplanting robotic arm based on previous research^31^, as shown in Fig. 5a,b.

The motor of the seedling claw adjusts the pushrod through the eccentric wheel to perform its opening and closing, as shown in Fig. 5a. The fixed plate is equipped with a proximity switch. When the eccentric wheel rotates, the controller receives the electric signal fed back by the proximity switch, providing it the seedling claw state. It then controls the force of the latter to properly grasp the sweet potato seedling. The sweet potato transplanting trajectory is in a pre-set plane. A transplanting mechanism with two degrees of freedom can follow various transplanting trajectories to meet the requirements of sweet potato transplanting. A sliding plate motor can drive the seedling claw to move up and down along the guide rail, and the rotary motor can drive it to rotate, as shown in Fig. 5b. In this design, the transplanting robot has two degrees of freedom, allowing it to reach different transplanting tracks. The side of the sliding plate is equipped with proximity switches. The signal from each one can correct the initial position of the transplanting and avoid the cumulative deviation during the whole process.

#### Influence of the seedling claw length on the transplanting operation

**Fig. 6:**
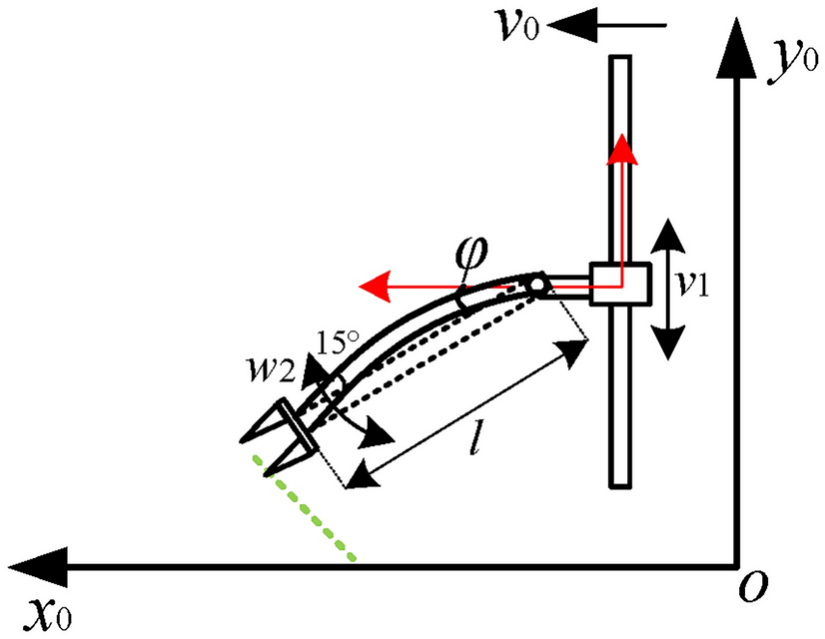
Transplanting machine arm model. *Note*: *v*_0_ represents the operating speed of the transplanting machine; *φ* is the initial angle of the seedling claw; *l* denotes the length of the seedling claw rod; *ω*_2_ is the angular velocity of the seedling claw; *v*_1_ is the sliding table movement speed.

The initial angle of the seedling claw (*φ*) and the length of its rod (*l*) affect the transplantation efficiency of sweet potato seedlings. Thus, they are considered as crucial structural parameters of the transplanting machine arm shown in Fig. 6.

**Fig. 7:**
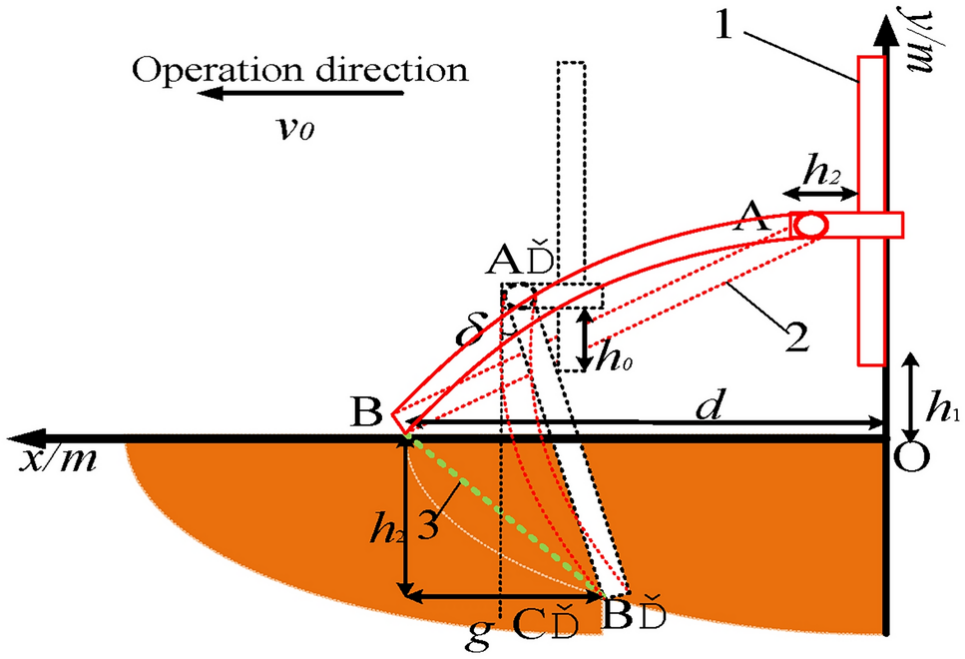
Analysis of the length and size of a claw bar. 1. Sliding Table 2. Seedling claw 3. Transplantation trajectory of sweet potato seedlings under different transplanting methods. *Note*: *h*_0_ is the shortest distance between the moving platform of the seedling claw and the bottom of the sliding table, *h*_1_ is the distance between the bottom of the sliding table and the top of the ridge, *h*_2_ is the maximum distance for transplanting sweet potato seedlings, *d* is the distance between the sliding table and the sweet potato seedlings, *g* is the maximum length of the sweet potato seedlings entering the soil, and *v*_0_ is the maximum operating speed of the transplanter.

The geometric dimensions of the transplanting machine arm are calculated based on the trajectory of the sweet potato seedling transplantation. Figure 7 shows the length and size of a claw bar, where the solid red line represents the initial state of the transplanting machine arm, the orange section represents its working space, and the dashed black line represents its final state after one *T*. It can be seen that the equipment operates at a speed of *v*_0_. After the transplantation cycle *T*, the mechanical arm moves from position *AB* to position *A*’*B*’ to perform a single transplanting of sweet potato seedlings.

The length of the seedling claw makes the sweet potato transplanting trajectory meet the following conditions in the working space of the transplanting manipulator:

For a sweet potato transplantation, the seedling claw point B’ and point B should be greater than the maximum depth of sweet potato transplantation (*h*_2_):

1 $$\left\{ {\begin{array}{*{20}l} {l\alpha \cos \delta \ge h_{0} + h_{1} + h_{2} } \hfill \\ {\delta = \arctan \frac{{h_{2} }}{{h_{0} }}} \hfill \\ \end{array} } \right.$$

where α is the safety factor.

To ensure that the horizontal distance between the robotic arm point *B*′ and point *B* is greater than the maximum length *g* of the sweet potato seedling into the soil, the installation position *d* of the transplanting robotic arm should be greater than the sum of the transplanter forward distance in a transplanting period *T*_0_ and the maximum length of sweet potato seedling into *g*:

2 $$d + \sin \delta l - v_{0} T \ge g$$

Equations (1) and (2) are combined, and parameters are then set according to the agronomic requirements and the actual size of the transplanting prototype. In particular, $\alpha = 0.8$,* h*_0_ = 0.05 m, *h*_1_ = 0.05 m, *h*_2_ = 0.15 m, *g* = 0.15 m, *v*_0_ = 0.35 m/s, and *T*_0_ = 1 s. This results in:

3 $$\left\{ {\begin{array}{*{20}l} {l \ge 0.396m} \hfill \\ {d \ge 0.5 - 0.7l} \hfill \\ \end{array} } \right.$$

Increasing the length of the seedling claw will increase the transplanting torque. Sweet potato transplanting is a high-frequency and long-term operation process. Increasing the operating torque will decrease the stability of the transplanter. Therefore, the minimum length of the seedling claw will be chosen in this paper. Find *l* = 0.4 m. Insert it to Eq. (3) result in $d \ge 0.224m$.

#### Impact of the initial angle of the seedling claw on the transplanting operation

**Fig. 8:**
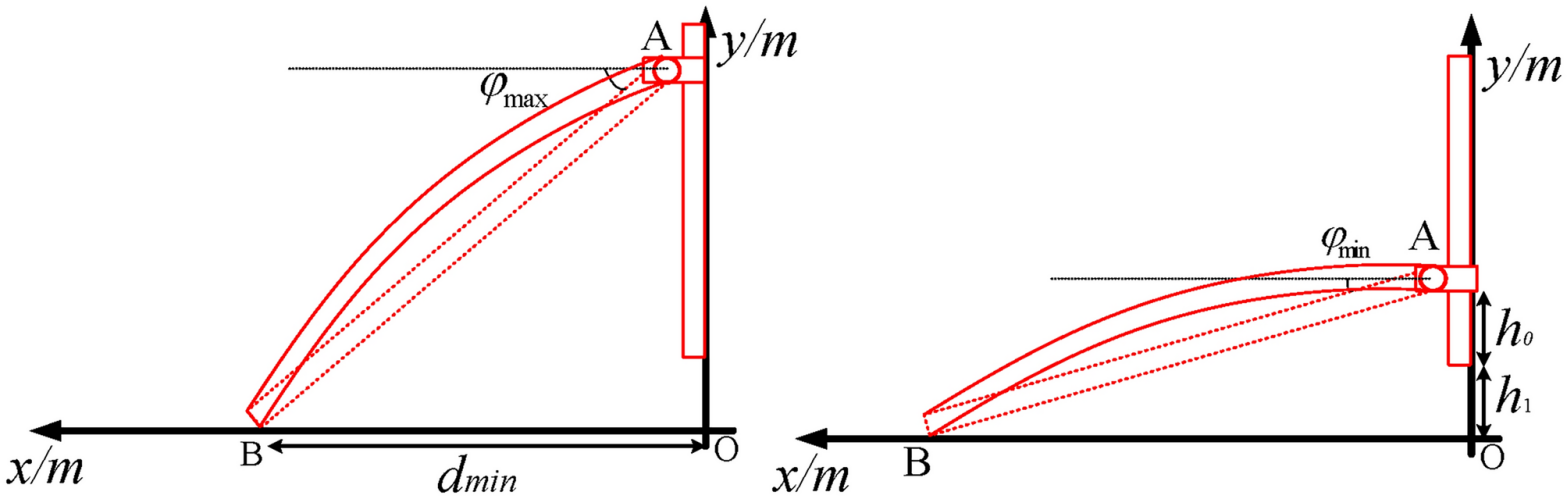
Analysis of the initial rotation angle of the transplanting manipulator. (**a**) Minimum angle of seedling claw; (**b**) Maximum angle of the seedling claw.

It can be seen from Fig. 8a that, for *OB* = *d*_min_, the initial angle of the seedling claw reaches its maximum value $\varphi_{\max } = ar{\text{c}} \sin \frac{{d_{\min } }}{l}$. In addition, it can be observed from Fig. 8b that, for *OA* = *h*_0_ + *h*_1_, the initial angle of the seedling claw reaches its minimum value $\varphi_{\min } = ar\cos \frac{{h_{0} + h_{1} }}{l}$. The range of the initial angle *φ* of the seedling claw is calculated by substituting the relevant parameter values, yielding 14.32° ≤ *φ* ≤ 55.93°. Note that the middle value of the initial angle is considered, that is *φ* = 35°.

## Control system design scheme

**Fig. 9:**
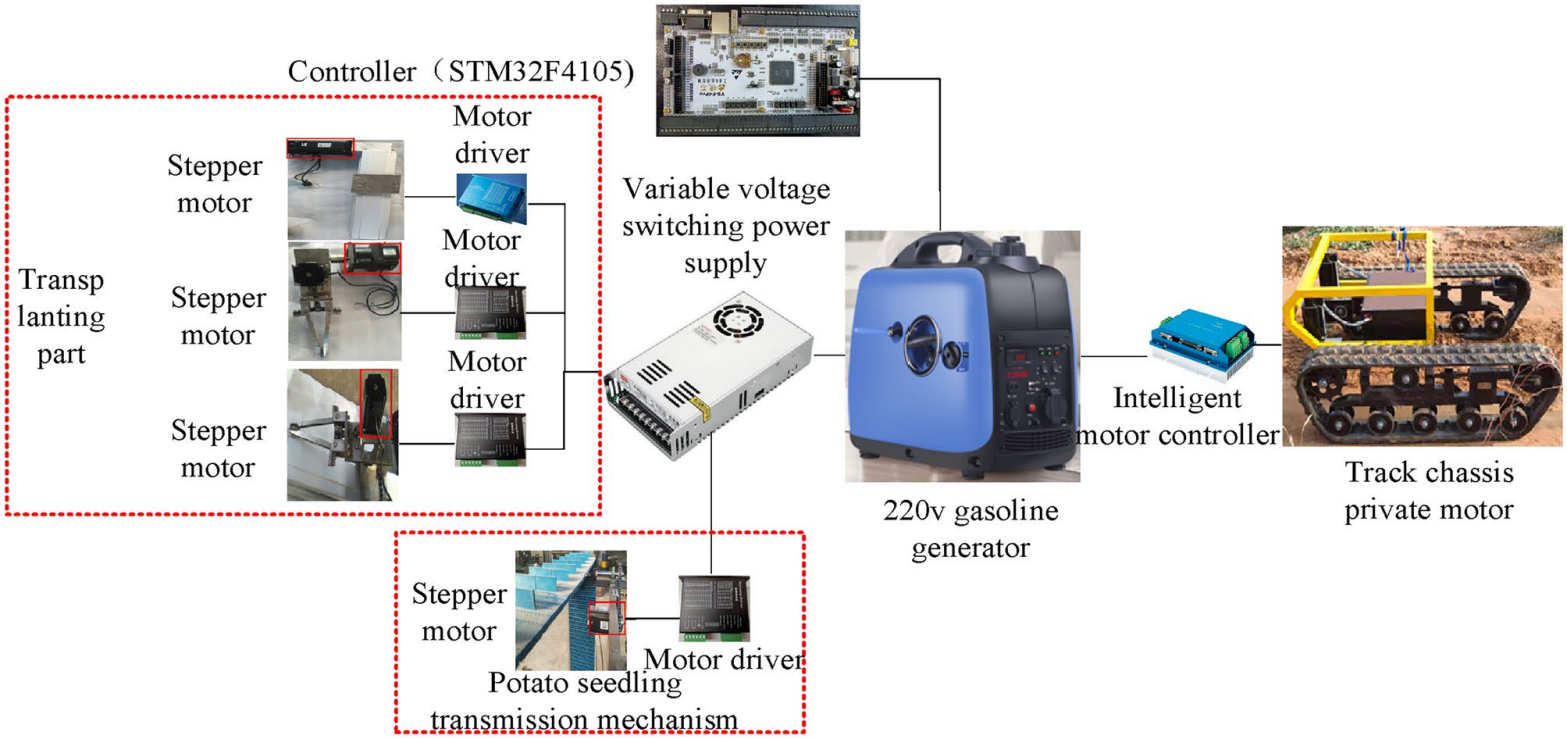
Analysis of the initial rotation angle of the transplanting manipulator. (**a**) Minimum angle of seedling claw; (**b**) Maximum angle of the seedling claw.

The control system scheme of sweet potato transplanting is shown in Fig. 9. The STM32F407 development board is used as the transplanting controller. It receives and processes the electrical signals returned by the sensor (proximity switch). In addition, it runs the corresponding control algorithm and then issues the control command to the drive element. The latter (e.g., closed-loop stepper motor and servo motor) receives the controller instruction and drives the executing element (e.g., seedling claw, sliding platform, crawler chassis, and potato conveyor belt) to perform potato seedling transmission and transplanting. To perform the correct seedling taking and transplanting action, timing requirements for the transmission mechanism, mechanical arm transplanting mechanism, and crawler chassis should be taken into consideration.

After manually pressing the start switch of the control box, the Shiloader starts the initialization process, promoting the seedling claw to perform the origin regression action. The potato seedlings conveyor belt starts to transmit the potato seedlings. When the potato seedling conveyor belt approach switch, slide table mobile motor approach switch, rotary motor approach switch, and seedling claw motor approach switch are all in a high-level state, the potato seedlings are transported to the designated location. In addition, the robotic arm seedling claw is in the initial position. Preparation for transplanting is then conducted. When the transplanting distance reaches a certain value, the controller sends the transplanting signal, the seedling claw is closed, the potato seedlings are taken, and the transplanting starts. At this time, the seedling conveyor belt approach switch, slide table mobile motor approach switch, rotary motor approach switch, and seedling claw motor approach switch are all at low-level state. The potato seedlings conveyor belt then starts to transmit the potato seedlings. The robotic arm seedling claw is in working condition. The return command will automatically open on the robotic arm after the transplanting action is completed. At this time, the potato seedling conveyor belt approach switch, slide table mobile motor approach switch, rotary motor approach switch, and seedling claw motor approach switch are all at high-level state again. This shows that the transplanter is ready for the next potato seedling transplanting.

## Impact of the soil on the transplanting performance

In order to further demonstrate the high effectiveness of the transplanting mechanism, the EDEM discrete element simulation software and the RECURDYN multi-body dynamics software were used to study the influence of the soil on the transplanting performance. A relevant study^32^ have shown that sandy loam is suitable for planting sweet potato. Therefore, this study adopts the sandy loam as the soil environment. A virtual prototype model was first developed in RECURDYN by importing the transplanting manipulator, sweet potato seedling, and soil covering model. The soil model was then built in EDEM. Afterwards, the sweet potato seedling, seedling claws, and covering disk in RECURDYN were imported into EDEM to conduct a co-simulation. As the leaves of the sweet potato seedling will not affect its position beneath the soil in the transplanting process, the sweet potato seedling is simplified as a slender cylindrical flexible body having a diameter of 4 mm and a length of 250 mm. Experiments were conducted to study the impact of the soil particles on different transplanting methods, as well as that of the soil moisture content on the transplanting trajectory. Moreover, the changes of the sweet potato seedling posture were studied. The parameters of potato seedlings are set as shown in Table 1^3^. In the experiment, the speed of the prototype and the initial rotation angle of the seedling claws were set to 0.2 m/s and 35°, respectively. The simulation environment is shown in Fig. 9.

**Table 1:** Sweet potato seedling parameters.

Parameter	Length/mm	Diameter /mm	Density/kg m^3^	Poisson’s ratio	Shear modulus/pa
Value	2	5	1152	0.3	1.81e + 5

### Changes of the position posture

**Fig. 10:**
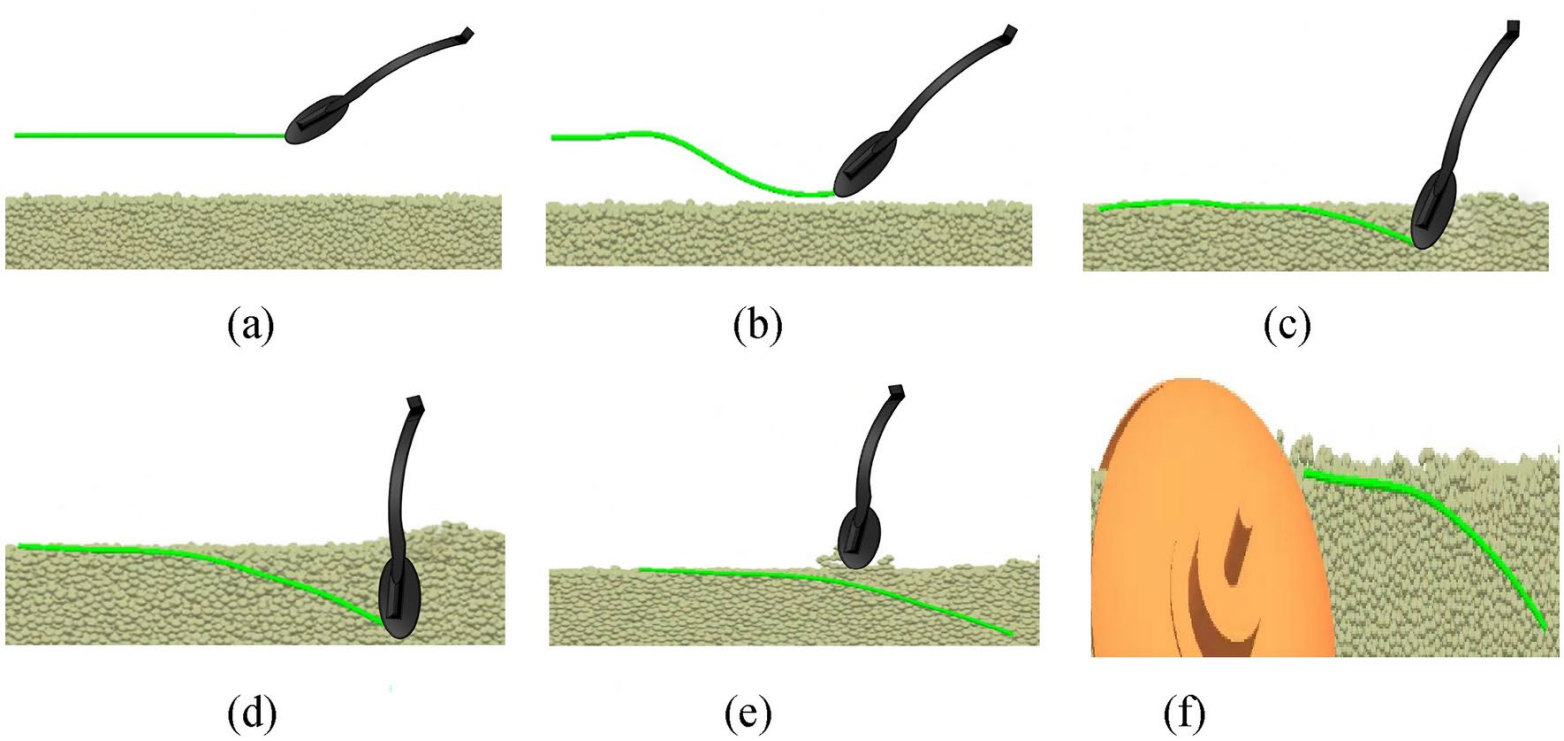
Sweet potato seedling pose changes. (**a**) 0 s; (**b**) 0.12 s; (**c**) 0.43 s; (**d**) 0.57 s; (**e**) 0.85 s; (**f**) 1.9 s.

The soil type and transplanting method are set to sandy loam and inclined planting, respectively. The sweet potato transplanting test is then conducted. The detailed process is shown in Fig. 10.

When the sweet potato seedling is not clamped (i.e., at 0 s), it is placed on the horizontal pallet, and it is ready to be transplanted. At 0.12 s, the seedling claws are used to clamp the sweet potato seedling, adjust their posture, and insert them diagonally into the soil. At 0.57 s, the root of the sweet potato seedling reaches the lowest point of the transplanting process. The claws of the seedling are opened and the sweet potato seedling falls into the ridge. At 0.85 s, the robotic arm leaves the soil ridge, and the soil returns to the pit formed by transplanting. From 0.85 to 1.9 s, the soil covering machine moves near the position of the sweet potato seedling to fill the pits created by the transplanting. At this time, the position of the sweet potato seedling is inclined planted and the robot arm completes one sweet potato transplanting cycle. It is deduced that the sweet potato seedling is slightly out of the ridge surface and its posture in the ridge meets the agronomic requirements of transplanting.

### Impact of the soil particles on different transplanting methods

**Fig. 11:**
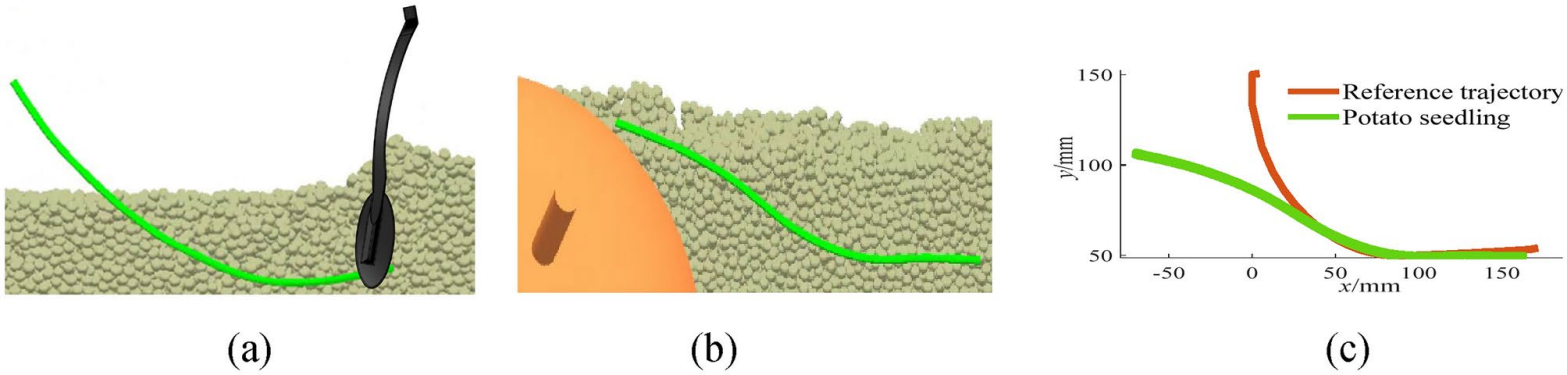
Horizontal transplanting of sweet potatoes. (**a**) 0.45 s; (**b**) 1.9 s; (**c**) Transplanting effect comparison.

**Fig. 12:**
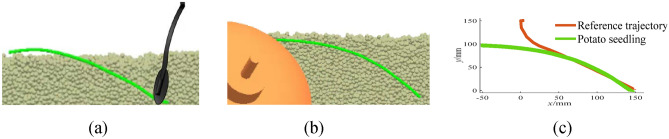
Inclined transplanting of sweet potatoes. (**a**) 0.45 s; (**b**) 1.9 s; (**c**) Transplanting effect comparison.

The soil type is set to sandy loam, and the influence of the soil particles on the horizontal transplanting and inclined planting trajectories is studied. The obtained results are shown in Figs. 11 and 12.

It can be seen from Fig. 11 that the soil particles significantly affect the horizontal transplanting trajectory. This is due to the fact that the horizontal transplanting has two trajectories. The first half trajectory is a circular arc while the second one is a horizontal straight line. When the sweet potato seedling moves to the second half, it is stretched by the seedling claws, and the track of the first half of the sweet potato seedling is extruded and rubbed with soil particles, which results in large deformation. However, the second half remains horizontal. Sweet potato caking mainly occurs in the second half of the straight line, and the first half of the arc is mainly used to transplant the sweet potato seedling into the soil at an appropriate depth. Therefore, the trajectory still meets the agronomic requirements, and there is no need to adjust the transplanting track. It can be seen from Fig. 12 that the soil particles have a slight impact on the inclined transplanting trajectory. This is due to the fact that the transplanting trajectory of the inclined planting method is an inclined line, and the stretching action in the latter part of the transplanting track does significantly affect the first half of the track.

## Field experiment

### Test conditions

Relevant studies^15^ show that two experimental factors, operation speed and showing length of feeding, have a great influence on the seedling planting performance, but their interaction effect is small and can be ignored. Therefore, two-factor three-level orthogonal tests are carried out to study the influence of the above factors on the qualified rate of transplanting and the leakage rate.

According to the “Technical Specification for Production of Sweet Potato in Shanxi Province”, the qualified rate of sweet potato transplanting is defined in detail. The qualified rate of sweet potato transplanting mainly includes three aspects: the insertion angle of the sweet potato seedling, the planting depth, and the length of the seedling into the soil. The insertion angle of the sweet potato seedling refers to the angle between the root of the sweet potato seedling into the soil and the horizontal plane. The acceptable range of the inclined planting method is 30°-35°, and the acceptable range of the horizontal planting method is 0°–5°. The planting depth refers to the deepest point of the root of the sweet potato seedling into the soil. It is 8–10 cm for the inclined transplanting method and 5–6 cm for the horizontal planting method. A reasonable range for the length of the sweet potato seedling in the ridge is 15–18 cm. When meeting the above evaluation criteria, it can be judged that the sweet potato transplanting operation is qualified.

**Fig. 13:**
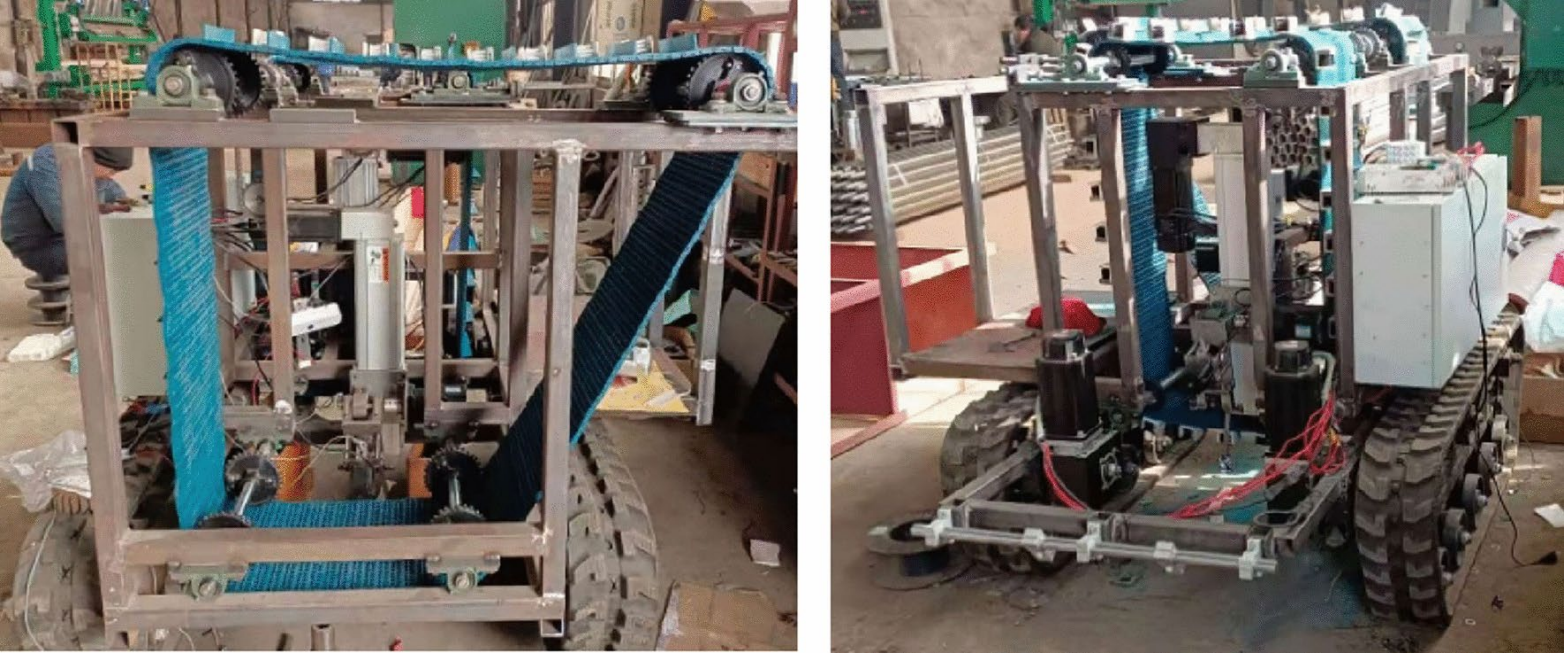
Processing site of sweet potato transplanting machine (**a**) Front view (**b**) Rear view.

According to the designed 3D drawings of the transplanter, complete the processing, assembly, debugging, and testing of the relevant components. The processing site of the transplanter is shown in Fig. 13.

The test site is the sweet potato planting base in Tai’an City, Shandong Province. The test is in April with the sandy loam soil type, and the parameters of the entire machine are shown in the Table 2. The moisture content of the soil ridge is between 19 and 22% measured by TDR 150 (Nanjing Ming Ao Instrument Equipment Co., LTD). According to the agronomic standards of sweet potato soil ridges, the ridge height is 250 mm, the ridge top width is 200 mm, and the ridge bottom width is 600 mm. The ridge planting was used for planting without mulching, and the planting spacing was about 300 mm. The seedling samples used in the experiment were the “Yanshu 25”. The average seedling length was 257 mm, the seedling diameter was 5.1 mm,the average distance of nodules was about 32 mm, and the number of leaves was 8–12 pieces.

**Table 2:** Structural parameters of sweet potato transplanter.

Item	Value
length × width × height/(mm × mm × mm)	1600 × 1200 × 1400
Working width/(mm)	900
Matching power/(kw)	5
Most suitable row spacing/(mm)	900
Ridge height/(mm)	> 300
Distance between hills/(mm)	> 200
Forward speed/(m s^−1^)	0.1–0.4
The length of the seedling claw (m)	0.4
The initial angle of the seedling claw (°)	35°

### Test plan

In the experiment, each testing group is made of 200 plants. And the qualified rate of transplanting *Y*_1_ and the leakage rate *Y*_2_ in each group were tested respectively, and the average value was taken. The test factors and levels are shown in Table 3.

**Table 3:** Experimental factors and levels.

Levels	Experimental factors	
	Operation speed/m s^−1^	Showing length of feeding/mm
1	0.1	40
2	0.2	50
3	0.3	60

The qualified rate of transplanting is calculated as:

4 $$Q = \frac{{n_{0} }}{N}$$

where *n*_0_ is the qualified number of potato seedlings transplanting and *N* is their total number.

The leakage rate is:

5 $$M = \frac{{n_{1} }}{N} \times 100\%$$

where *n*_1_ is the number of potato seedlings.

### Data processing

The test results and range analysis are shown in Table 4. It can be seen that the effect of operation speed and the length of feeding on inclined transplanting and horizontal transplanting is consistent. The range analysis shows that *A*_1_*B*_2_ is the best combination of the experimental factors for qualified rate, and the primary and secondary order of factors is operation speed > length of feeding. *A*_1_*B*_3_ is the best combination of the experimental factors for the leakage rate, and the primary and secondary order of factors is the length of feeding > operation speed.

**Table 4:** Test scheme and results.

Test number		Experimental factors		Evaluation index			
		Operating speed *A*	Length of feeding *B*	Inclined transplant qualified rate *Y*_1_	Horizontal planting qualified rate *Y*_1_	Inclined transplant leakage rate *Y*_2_	Horizontal planting leakage rate *Y*_2_
1		1	1	96.6	97.5	2.9	3.0
2		1	2	97.2	97.8	2.2	2.1
3		1	3	96.5	96.5	2.4	1.6
4		2	1	94.3	96.3	3.5	3.7
5		2	2	96.5	96.6	2.3	2.5
6		2	3	93.8	94.8	2.1	2.8
7		3	1	93.2	95.1	4.3	3.6
8		3	2	95.3	95.0	3.1	2.6
9		3	3	94.0	94.4	2.6	2.3
Inclined transplant qualified rate *Y*_1_	*k*_1_	97.30	96.37	Horizontal planting qualified rate *Y*_1_	*k*_1_	96.70	94.67
	*k*_2_	96.00	96.57		*k*_2_	94.93	96.47
	*k*_3_	94.93	95.30		*k*_3_	94.30	94.80
	*R*	2.4	1.3		*R*	2.4	1.8
	Primary and secondary factors	*A* > *B*		Primary and secondary factors	*A* > *B*		
	Optimal combination	*A*_1_*B*_2_		Optimal combination	*A*_1_*B*_2_		
Inclined transplant leakage rate *Y*_2_	*k*_1_	2.30	3.60	Horizontal planting leakage rate *Y*_2_	*k*_1_	2.51	3.50
	*k*_2_	3.00	2.35		*k*_2_	2.63	2.56
	*k*_3_	2.93	2.23		*k*_3_	3.40	2.37
	*R*	0.7	1.4		*R*	0.9	1.2
	Primary and secondary	*B* > *A*		Primary and secondary	*B* > *A*		
	Optimal combination	*A*_1_*B*_3_		Optimal combination	*A*_1_*B*_3_		

Variance analysis of seedling operation quality indexes is shown in Table 5. For the index of leakage rate, the horizontal transplanting operation speed and the length of feeding have a significant effect (*P* < 0.05); Inclined transplanting operation speed have a significant influence (0.01 ≤ *P* ≤ 0.05) and the length of feeding have extremely significant influence (*P* < 0.01). For the index of the qualified rate, the operation speed of horizontal transplanting is extremely significant, and the influence of the length of feeding is significant;the operation speed of inclined planting and the length of feeding have a significant influence.

**Table 5:** Variance analysis of planting seedling quality indexes.

Index	Sources of variation	MS	*F* value	*P* value	Index	Sources of variation	MS	*F* value	*P* value
Horizontal planting qualified rate *Y*_1_	Operation speed *A*	9.28	11.4	0.022	Inclined planting qualified rate *Y*_1_	Operation speed *A*	8.43	23.3	0.006
	Length of feeding *B*	6.04	7.4	0.045		Length of feeding *B*	2.78	7.68	0.043
Horizontal planting leakage rate* Y*_2_	Operation speed *A*	1.35	7.14	0.048	Inclined planting leakage rate *Y*_2_	Operation speed *A*	0.89	7.75	0.042
	Length of feeding *B*	2.58	13.6	0.016		Length of feeding *B*	3.34	28.9	0.004

To analyze the comprehensive effects of operating speed and the length of feeding on the quality of inclined transplanting and horizontal transplanting, this paper adopts a comprehensive combined evaluation method to find the best parameter combination to optimize the results of the test and increase the productivity to the evaluation index.

The productivity index is related to the operating speed. When the operating speed is 0.1 m/s, 0.2 m/s, and 0.3 m/s, the productivity is 20 pcs/min, 40 pcs/min, and 60 pcs/min respectively.

Three membership index models are established shown in Eq. (4). The fuzzy relationship matrix *R*_*r*_ is formed by the membership value as shown in Eq. (5)^33–35^ Determine the weight distribution matrix *P* = [0.33 0.33 0.33]. The fuzzy comprehensive evaluation value set *W* is determined by the fuzzy matrix *R*_*r*_ and the weight distribution matrix *P*, where *W* = *R*_r_*P*, and the comprehensive scoring results are shown in Table 6.

6 $$\left\{ {\begin{array}{*{20}c} {R_{in} = = \frac{{Y_{in} - Y_{imim} }}{{Y_{i\max } - Y_{i\min } }} \times 100\% (i = 1,3;n = 1,2,...,9)} \\ {R_{in} = = \frac{{Y_{i\max } - Y_{in} }}{{Y_{i\max } - Y_{i\min } }} \times 100\% (i = 2;n = 1,2,...,9)} \\ \end{array} } \right.$$

7 $$R_{r} = \left[ {\begin{array}{*{20}c} {r_{11} } & {r_{12} } & \ldots & {r_{13} } \\ {r_{21} } & {r_{22} } & \cdots & {r_{23} } \\ {r_{31} } & {r_{32} } & \cdots & {r_{33} } \\ \end{array} } \right]$$

**Table 6:** Results of comprehensive evaluation.

Test	Membership values of qualified rate* R*_1n_		Membership values of leakage rate* R*_2n_		Membership values of productivity* R*_3n_		Score* W*	
	Inclined planting	Horizontal planting	Inclined planting	Horizontal planting	Inclined planting	Horizontal planting	Inclined planting	Horizontal planting
*A*_1_*B*_1_	0.79	0.94	0.59	0.3	0	0	0.42	0.35
*A*_1_*B*_2_	1	1	0.95	0.78	0	0	0.59	0.52
*A*_1_*B*_3_	0.80	0.59	0.86	1	0	0	0.51	0.48
*A*_2_*B*_1_	0.26	0.51	0.36	0.087	0.5	0.5	0.34	0.34
*A*_2_*B*_2_	0.87	0.72	0.91	0.60	0.5	0.5	0.67	0.56
*A*_2_*B*_3_	0.13	0.061	1	0.48	0.5	0.5	0.49	0.31
*A*_3_*B*_1_	0	0.18	0	0	1	1	0.33	0.36
*A*_3_*B*_2_	0.56	0.13	0.45	0.53	1	1	0.60	0.52
*A*_3_*B*_3_	0.211	0	0.77	0.69	1	1	0.59	0.51

From Table 6, it can be seen that the optimal combination of horizontal transplanting method and inclined transplanting method is *A*_2_*B*_2_, that is, the transplanting operation speed is 0.2 m/s, and the showing length of feeding is 50 mm.

### Verification test

**Fig. 14:**
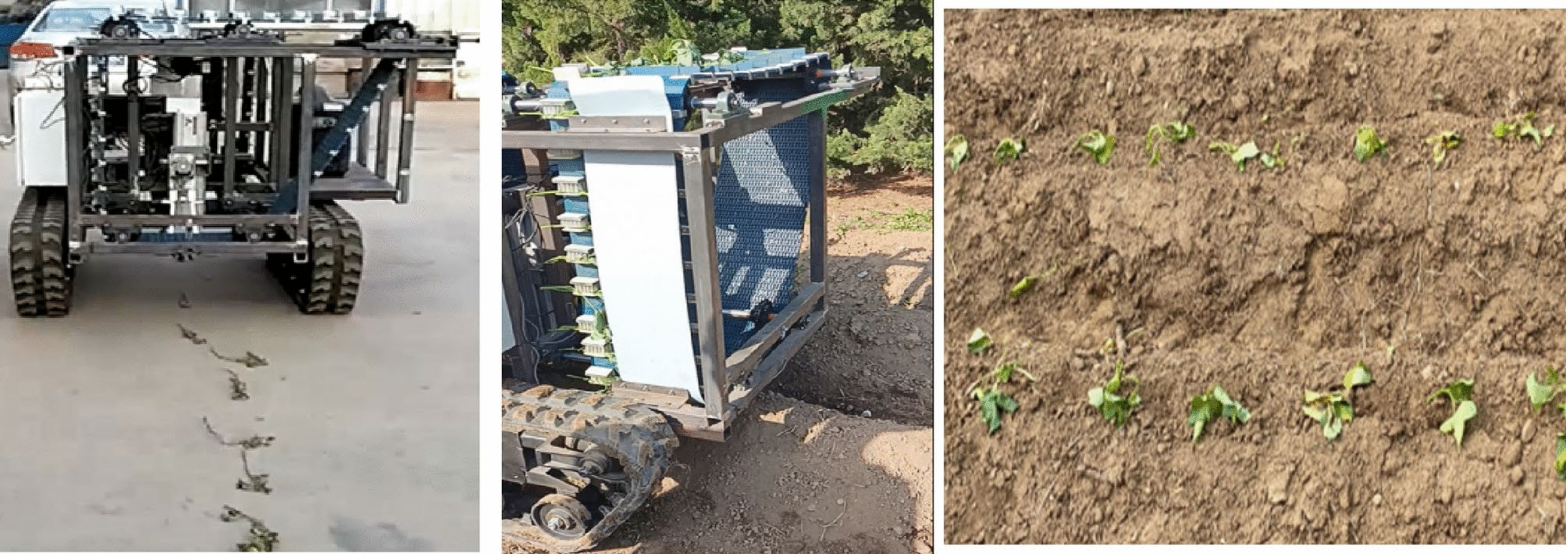
Sweet potato transplanting experiment (**a**) No-load test (**b**) Field test (**c**) Transplanting effect.

To the optimal combination scheme, a verification test was carried out. The test is shown in Fig. 14. The test results show that in the optimized sweet potato planting operation, the inclined planting method has a qualified rate 96.8%, a leakage rate 2.1%, and productivity index is 40 per minute. Meanwhile, the horizontal planting method has a qualified rate 96.9%, a leakage rate 2.4%, and a productivity index is 40 per minute. The optimized operation quality index of seedling planting is better than before.

### 5.5 Discussions

It can be seen from Tables 4 and 6 that the transplanting speed is the main factor affecting the transplanting success rate. This is due to the fact that, when the transplanting is too fast (i.e., its speed is greater than 0.2 m/s), the end of the claw throws a larger hole in the soil, which is not conducive to its reflux and to the fixation of naked seedlings, leading to the deformation of the potato seedling position after transplanting. This affects the transplanting quality. Thus, the shape of the seedling claw should be improved and the soil covering mechanism should be optimized. In addition, the length of feeding is the main factor affecting the seedling leakage rate. This is due to the large difference in the root shape of sweet potato naked seedlings, which can easily result in the seedling leakage phenomenon when the feeding length is small. Therefore, to ensure a correct seedling claw, a higher length of feeding is required.

It can be seen from the results of the simulation and field tests that the success rate of the sweet potato level transplanting is higher than that of the oblique planting. This is due to the fact that the oblique planting has larger depth and a seedling claw into a hole volume. In addition, when the seedling claw changes its trajectory during transplanting, the lower section, affected by the shape of the seedling claw, may bend, which affects the transplanting quality. Such issues could be prevented by ensuring that the trajectory of the seedling claw is consistent with the seedling shape during the final horizontal phase of the transplanting motion.

In the field test, the highest operation speed is equal to 0.2 m/s. This is due to the fact that the operation speed of the transplanting is affected by the manual seeding speed. In addition, the workers should also drive the transplanting forward. Therefore, the efficiency is low. Consequently, to increase the operation speed, transplanting machines for 2–4 lines simultaneously should be developed.

To further demonstrate the high effectiveness of the developed transplanting mechanism, a comparison is conducted with the key parameters of popular transplanting machines that are currently available on the market as the obtained results are shown in Table 7. It can be seen that the transplanting success rate of the developed machine is greater than that of most of the existing products. This is due to the fact that the developed transplanting mechanism can adjust its transplanting trajectory. In contrast to single-degree-of-freedom mechanisms, which cannot flexibly modify their size to align with agronomic requirements and only approximate the desired transplanting trajectory, the proposed mechanism ensures a precise alignment. Consequently, it reaches a higher quality of potato seedling transplanting.

**Table 7:** Comparison of transplanting machine operation effectiveness.

Serial number	Transplanting mechanism form	Oblique planting Success rate (%)	Horizontal transplanting success rate (%)	Source
1	Chain clamp transplanting mechanism	97.7	–	Hu et al.^15^
2	Four-bar linkage mechanism	93.7	–	Li et al.^34^
3	Flexible disc	–	–	Wu et al.^8^
4	Four-bar linkage mechanism	–	92.0	MU et al.^17^
5	Robotic arm type	94.0	92.0	Liu et al.^30^
6	Robotic arm type	96.8	96.9	This paper

When the seedling claw grips the sweet potato seedling, it is easy to cause damage to the sweet potato seedling. Although the controller can control the force of the seedling claws through the angle of the stepper motor, it will still cause a certain degree of damage to the sweet potato seedlings and affect the production rate of potatoes. The solution is to use a bionic flexible mechanical claw or an adaptive mechanical claw, which can detect the size of the object to be clamped, and adjust the posture and strength of the fixture in real-time to achieve precise grasping.

## Conclusions

This article introduces a new sweet potato transplanting robot, which can complete various transplanting methods such as inclined and horizontal transplanting for sweet potatoes. The transplanter’s structure, mechanical-plant-soil interaction mechanism and field experiment are studied in detail. The transplanter can perform the actions of picking and transplanting sweet potatoes according to a preset trajectory and complete a variety of transplanting spacing. The agronomic requirements of different planting crops are different, however, the planting principles are similar. As a result, the proposed machine can also be used as a transplanting machine for other crops.

In the field test, the inclined transplanting method has a qualified rate of 96.8%, and the horizontal planting method has a qualified rate of 96.9%. It can be seen that the transplanting performance of the transplanting machine is ideal. This research not only provides practical tools for the sweet potato planting market but gives a reference for the innovation, development, or optimization of sweet potato planting machinery as well.

## Data Availability

The datasets and code generated during the current study are available in the Zenodo repository, https://doi.org/10.5281/zenodo.14637140.
